# Augmented Realities, Artificial Intelligence, and Machine Learning: Clinical Implications and How Technology Is Shaping the Future of Medicine

**DOI:** 10.3390/jcm9123811

**Published:** 2020-11-25

**Authors:** Gaby N. Moawad, Jad Elkhalil, Jordan S. Klebanoff, Sara Rahman, Nassir Habib, Ibrahim Alkatout

**Affiliations:** 1 Department of Obstetrics and Gynecology, The George Washington University Hospital, Washington, DC 20052, USA; rahman.sara@gmail.com; 2 Department of Obstetrics and Gynecology, Philadelphia College of Osteopathic Medicine, Suwanee, GA 30024, USA; jhelkhalil@gmail.com; 3 Department of Obstetrics and Gynecology, Main Line Health, Wynnewood, PA 19096, USA; jsk5068@gmail.com; 4 Department of Obstetrics and Gynecology, Francois Quesnay Hospital, 78200 Mantes-la-Jolie, France; dr.nassirhabib@gmail.com; 5 Campus Kiel, Kiel School of Gynaecological Endoscopy, University Hospitals Schleswig-Holstein, 24103 Kiel, Germany; ibrahim.alkatout@uksh.de

**Keywords:** surgery, artificial intelligence, machine learning, augmented reality

## Abstract

Technology has been integrated into every facet of human life, and whether it is completely advantageous remains unknown, but one thing is for sure; we are dependent on technology. Medical advances from the integration of artificial intelligence, machine learning, and augmented realities are widespread and have helped countless patients. Much of the advanced technology utilized by medical providers today has been borrowed and extrapolated from other industries. There remains no great collaboration between providers and engineers, which may be why medicine is only in its infancy of innovation with regards to advanced technologic integration. The purpose of this narrative review is to highlight the different technologies currently being utilized in a variety of medical specialties. Furthermore, we hope that by bringing attention to one shortcoming of the medical community, we may inspire future innovators to seek collaboration outside of the purely medical community for the betterment of all patients seeking care.

## 1. The Future of Medicine

In nearly all aspects of healthcare, virtual reality (VR), augmented reality (AR), artificial intelligence (AI), and machine learning (ML) are becoming more commonplace. Although this technology impacts all healthcare disciplines, its significance is paramount for surgical disciplines. The decision to undergo surgery, whether elective or emergent, causes intense emotion. Patients place immense trust in their surgeon, essentially placing their own life in another’s hands. As surgeons, operating on patients should never be taken for granted and we should continue to seek improvements and ways to provide safer care.

An important aspect to recognize is the huge mountain to climb in actually implementing safer care for patients. This safer care comes with the evolution of newer surgical techniques and technology. This evolution is paralleled in all other industries in the world due to the fast advancement of technology and AI. The evolution, specifically in healthcare, is much easier said than done. A massive learning curve presents that requires most current and future physicians to understand, implement, and analyze the results of this technology. What does that mean for physicians? The presence of specialized training for these new technologies must emerge quickly in order for the healthcare field to keep up with the growth of data available. Providing patients with more effective and safer practices is a consequence of this.

Change in surgery is fear-provoking; it is unknown and unpredictable. When laparoscopy was first introduced, it was thought to be a technological “trend” that would not survive the test of time. Now laparoscopy is considered the safer method whenever feasible for patients. Similarly, robotics is under scrutiny. However, all would agree, including critics, that the robotic surgical console provides surgeons with a level of technical ergonomics (7 degrees of freedom) that is absent from conventional laparoscopy [1]. Other factors are also greatly enhanced by robotic surgery, such as precision, flexibility of movement, and completion time. These improvements exist in robotics because of the advancement of different technologies including AR and AI.

## 2. Virtual and Augmented Reality in Medicine

VR consists of creating a simulation of a given scenario, as opposed to the alteration of an actual reality. Daily, this can be used in video games, design planning, and even simulated roller coaster rides. In healthcare, VR could include a cadaver to help learn anatomical structures and any preoperative imaging to help plan a procedure. VR training curriculums were created in order to allow both training and practicing surgeons to a safer operating room (OR) experience for their patients [2]. VR can also be incorporated into a surgeon’s preoperative planning, utilized in unison with AI algorithms, to help virtually map a procedure [3]. VR is not limited to the OR; VR is being incorporated into occupational therapy to help stroke patients recover [4]. VR-based rehab is a proven tool that creates specific scenarios for patients, allowing them to have targeted treatments for their particular recovery level and deficits [4].

**Figure 1:**
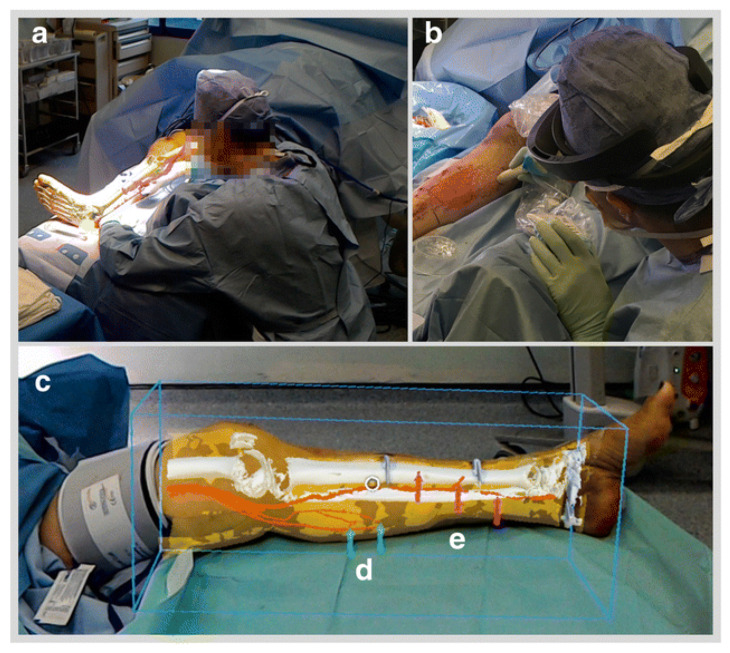
Use of HoloLens augmented reality (AR) goggles during operation in order to increase the accuracy of incision during a lower limb procedure. (**a**): remote view of AR through HoloLens. (**b**): Confirmation of perforator vein location. (**c**): Bound box overlay with (**d**) arrows pointing at medial sural veins and (**e**) arrows pointing at posterior tibial perforators.

From the ever-growing increase in medical complexity, there was born a need for technology to go beyond mere simulated reality. Augmented reality (AR) was the answer to this problem. With AR there is no created scenario; instead, an actual event is being altered in real time, which has significantly enhanced robotic surgery. AR can work in parallel with a telemanipulation system in order to optimize the visual field of the operating surgeon. AR is currently being utilized to overlay key anatomic landmarks during live surgery to optimize patient safety [5]. For example, the preoperative imaging studies of a patient can be superimposed on to the surgical field and highlight structures using markers. Figure 1 shows how AR was used to overlay a preoperative CT image in an extremely accurate fashion on a patient’s lower leg. Specifically, the HoloLens was used.

This technology allows the surgeon to have increased accuracy during the operation. Targeted guided surgery (TGS) involves a planned approach to a given procedure based on preoperative images. Once the surgery has begun, TGS implements real-time guidance imagery, using AR, that is shown on the endoscope using the predetermined plan [6]. In robotic surgery, the operator has a lack of tactile feedback. AR can partially fill this gap in feedback by enhancing the visual field of the surgeon. For example, the use of AR in real time can help the surgeon visualize how much cancerous tissue remains in the area of interest [7]. The application of AR in robotic surgery goes beyond detecting cancerous tissue, as seen above, and will continue to advance in order to provide the operator with an optimal visual field. The advances made in VR and AR have allowed for even more complex tasks to be handled by machines. Both technologies are currently used by telemanipulation systems in minimally invasive surgeries. Patient risk of post procedure complications has been reduced with the use of these visual technologies and robotic surgery. One surgeon performed robotic-assisted laparoscopic radical prostatectomy (RALP) on 200 patients over the course of 5 years. Over time, the necessity for blood transfusions and the presence of major complications were recorded in response to the procedure and were shown to be significantly less, and sometimes close to 0%, in patients [8]. These results can provide a great vision into the potential future reduction in major complications in procedures for patients. Figure 1 provides a simple demonstration of the use of AR in surgery through the use of the HoloLens [9].

## 3. Artificial Intelligence, Machine Learning, and Deep Learning

With the ever-growing supply of healthcare data, AI algorithms have aided in data organization, synthesis, and analysis [10]. Tasks that would take hundreds of person-hours to complete can be done almost instantaneously by machines with minimal deficiencies, mistakes, or bias. AI has likely had the most profound impact in healthcare by revolutionizing the electronic health record (EHR). AI is now able to expand differential diagnoses, recognize early warning signs of patient morbidity or mortality, and identify abnormalities in imaging studies or images [11,12]. Once an algorithm has recognized a pathologic condition through training, newly generated images based on a cache of previously learned images are applied to educating and testing [9]. AI is utilized to enhance learning for trainees and medical students [12]. This enhancement comes from providing more “realistic” OR situations and even creates previously unseen imaging for the students to assess. AI can also remove the risk of a student making a mistake in their first surgical experience by preparing them with real-time OR situations using AI (in a combination of VR).

**Figure 2:**
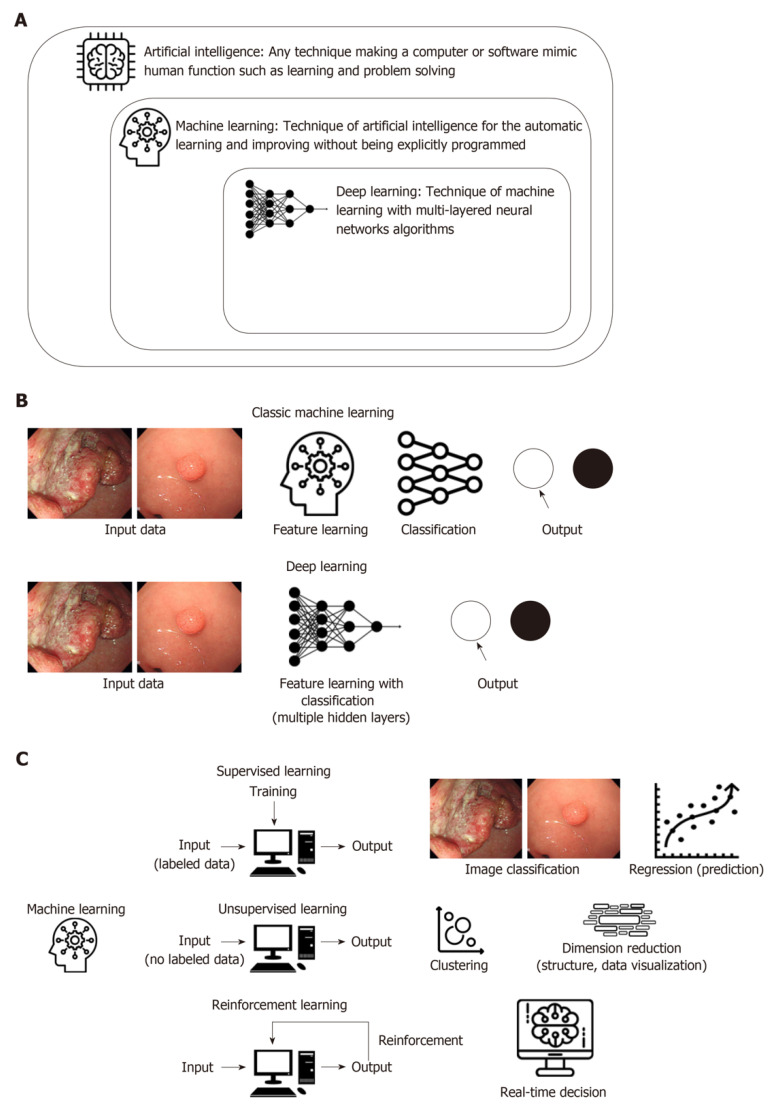
Representation of the processing methods of different types of artificial intelligence (AI). (**A**): Defining AI, ML and DL. (**B**): Difference in how ML and DL algorithms process information (**C**): Demonstrating the different types of learning.

Under the umbrella of AI exists machine learning (ML), which consists of teaching a given dataset as a curriculum to a machine. With that dataset programmed into the machine’s neural network, new tasks can then be completed through the integration of the learned system into a new task [13]. The new task is first separated into its component parts and then the learned algorithm is applied as a linear regression until each individual component is solved. This differs from deep learning (DL), which is considered a sub-category of ML. Within DL, a computerized neural network is taught multiple datasets and a layered regression model is applied to a given task using these multiple datasets. To help imagine the regression model created by DL, think of the normal linear regression model, y = mx + b. There is only one variable considered here. Adding one or two or even three more variables would still be fairly simple. However, imagine a layered regression model including thousands if not hundreds of thousands of variables. This makes the DL neural network a very complex but useful tool. The standalone difference between ML and DL is that with DL, the machine completes a complex decision-making algorithm and there is not a traceable path as to how the machine reached its conclusion [14]. For example, deep learning is needed for complex tasks such as a fully autonomous surgery. However, in order to have a fully autonomous surgery, normal machine learning algorithms are needed in unison with deep learning algorithms to function efficiently. Figure 2 below provides simple definitions for the different types of AI and also compares the processing steps and types of learning used to teach the ML algorithms. [15].

## 4. Current Limitations of Artificial Intelligence in Surgery

Unfortunately, despite these newly developed neural networks allowing machines to complete complex tasks, the environments in which they function are static. When any change is introduced into the system outside of what has been learned, the neural network is not efficient at adapting. There are currently too many unknowns and potential changes that occur during surgery that it would not be safe nor effective to have a completely automated surgery.

To combat this issue, outlier detection algorithms are used in order to recognize an unknown or a problem that the algorithm cannot accomplish. That unknown is then taught to the algorithm so that the next time it is seen, the algorithm knows how to react. This process is crucial to reach a fully autonomous surgery algorithm. The algorithm must go through many surgical scenarios over and over again in order to constantly decrease the number of outliers. Unfortunately, there will never be a way to eliminate all the outliers and the algorithm will always have to be slightly altered in order to navigate challenges.

Additionally, there are numerous shortcomings with the hardware (physical pieces) of the current neural networks. However, even if the right hardware existed, there currently are too many restrictions on gathering patient data to allow the neural networks to properly learn. The ability of a neural network to learn is largely dependent on the amount of data points the system is given. In other areas of neural networking, like autonomous vehicles, datasets are widely available and easily gathered. The issue in medicine stems from the patient–physician relationship. Large datasets have not yet been created out of concern for breach of confidentiality, which exists between a single patient and provider [16]. In order for us to move towards autonomous surgery, we need our robotic surgical algorithms to first be able to learn. This requires access to a vast amount of real patient data that would allow the algorithm to have enough surgical information to implement certain techniques (after series of training and testing). In simple terms, the more access to patient data, imaging, real-time OR data, and even virtual OR data we have, the more algorithms can be taught. Without easy access to this data, the algorithm will be restricted in its ability to perform more complex tasks in healthcare.

## 5. Where We Are

We are not far from the first automated procedure. Already in orthopedics, robotic surgical systems are being used to cut bone with unparalleled precision [17]. Additionally, automated machines have proven to effectively suture as well as, or better than, surgeons with up to 5 years of training [13]. Fully autonomous procedures can be done today on fixed anatomical structures such as the eye and bone. However, the challenge comes when attempting a soft tissue surgery with constant moving structures. A bone is set in place and will experience very little movement during a procedure. On the other hand, the small intestines can be easily manipulated and could change position which makes the use of autonomous surgery tougher. Even if this position change is 2 mm, this could drastically jeopardize the task execution by the machine. In these cases, markers may need to be implemented on the soft tissue structures in order for the algorithm and machine to constantly track the structure(s).

Cost is the ongoing issue facing healthcare facilities in implementing any technological advancement with regard to automated procedures. Challenges surgeons have faced for many years such as fatigue, burnout, and tremor can all be decreased with this advancement, which would be beneficial for the healthcare industry. Automation will allow surgeons to do what they are best at with greater confidence, while making the best decision for the care of the patient.

Another rate-limiting step for automated surgical procedures is the regulation placed on protected health information, rightfully so. We must find a way to overcome this barrier and begin safe, confidential data sharing so that machines can begin to learn. Although it may seem far-fetched and unattainable, we must remember how laparoscopy was first viewed and how it has now become a staple of our surgical treatments. Google, Intuitive, Microsoft, Storz, and Olympus are some companies that could work towards bridging this gap of engineering and healthcare in order to safely and effectively break another barrier to improve healthcare as we know it.

Engineers are not the only ones that need to benefit from technological advancements. VR and AR were not created to exist in a silo for engineers. This technology, with the right team and handling, can advance any and every industry. The major key in taking this step forward in healthcare is establishing this crucial relationship. In the engineering world, this technology already exists and flourishes with the right amount of accessible data. For this reason, healthcare professionals and engineers must work together towards a common goal. If the two disciplines can work together, the limits and obstacles can and will be surpassed and the patient, who is the number one priority, can receive the care they deserve consistently.
